# Clinical significance of intraventricular gradient during effort in an adolescent karate player

**DOI:** 10.1186/1476-7120-5-39

**Published:** 2007-11-19

**Authors:** Carlos Cotrim, Ana G Almeida, Manuel Carrageta

**Affiliations:** 1 Cardiology department, Garcia de Orta Hospital, Almada, Portugal; 2 Cardiology department, Santa Maria Hospital, Lisboa, Portugal; 3 Lisboa University Medical School, Lisboa, Portugal

## Abstract

The authors report the case of a 16-year-old boy who practices karate, who underwent medical evaluation because of atypical chest discomfort, related to strenuous effort. The ECG and echocardiogram findings were normal. The young boy did a treadmill stress test which was positive for myocardial ischemia. Late during the investigation, he underwent treadmill stress echocardiography, during which he developed intraventricular gradient of over 130 mmHg with end-systolic peak and systolic anterior movement (SAM) of the mitral valve. These echocardiographic findings were not present at rest and disappeared shortly after termination of exercise. The authors discuss the significance of this event. This leads us to advise withdrawal from participation in competitive sport according to the recomendations of the European Society of Cardiology. A possible role of exercise stress echo for intraventricular pressure gradient assessment in symptomatic athletes with structurally normal hearts is suggested.

## Background

The development of significant intraventricular gradients during exercise is rare, usually in association with left ventricular hypertrophy [1,2]. In 23-year-old athlete [3], in an 20-year-old professional soccer player [4], and in a 79 year-old man we performed exercise echocardiography [5], and during the exercise [6] we unespectedly detected significant (greater than 50 mmHg) intraventricular gradient and systolic anterior movement of the mitral valve. In the case presented here, we describe an adolescent karate player that developed the same phenomenon.

## Case report

We describe the case of a 16 year-old caucasian male, with a four-hour weekly training schedule, who complained of occasional chest disconfort on strenuous exercise. There was no relevant personal history and no family history of sudden death or heart disease were reported.

**Figure 1:**
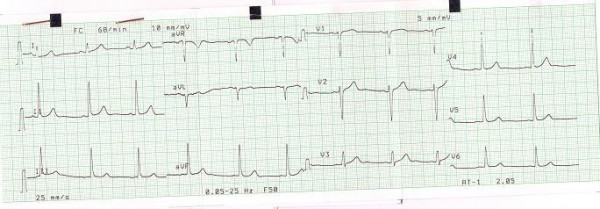
Normal ECG.

**Figure 2:**
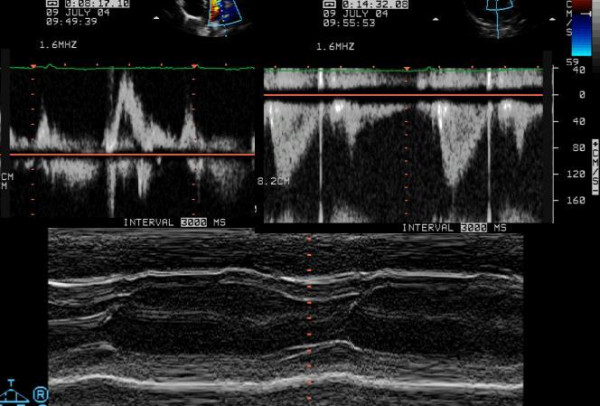
Normal echocardiogram without left ventricular hypertrophy.

**Figure 3:**
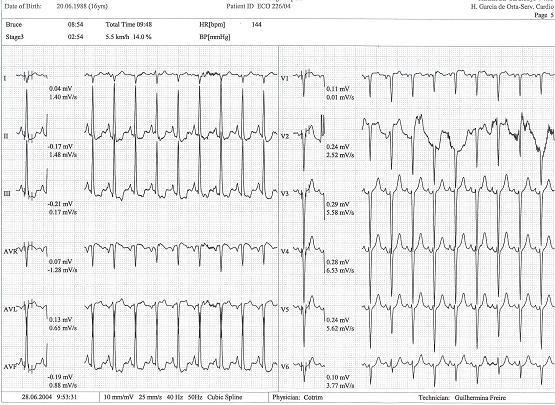
Exercise test with alteration in ST segment in DII, DIII and avF.

Physical examination revealed normal cardiac auscultation and normal radial, carotid and femoral pulses. The twelve lead electrocardiogram was normal (Figure 1). The echocardiogram was also normal (Figure 2, see Additional file 1), with no left ventricular hypertrophy; left ventricular end-diastolic diameter was 41 mm, septum and posterior wall were 7 and 6 mm respectively, and the left ventricular outflow tract was 17 mm. No abnormalities were found in the mitral valve of subvalvular apparatus. Treadmill exercise test was performed following the Bruce protocol that was considered positive for to myocardial ischemia (Figure 3).

**Figure 4:**
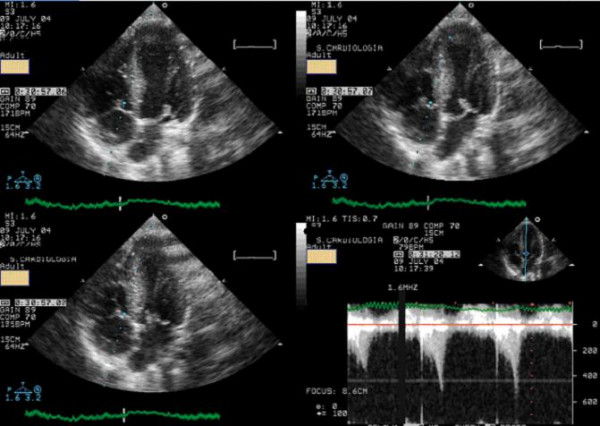
At peak exercise systolic anterior movement of mitral valve and significant intraventricular gradient was detected.

The patient underwent an exercise stress echocardiograpgy, and during the exam developed a significant intraventricular gradient of 130 mmHg with an end-systolic peak and systolic anterior motion of the mitral valve (Figure 4, see Additional file 2), which disappeared in the first minute of recovery period. There was no fall in blood pressure at the end of the test. The exercise stress echocardiogram was negative for myocardial ischemia.

## Discussion and conclusion

It has long been know that small intraventricular gradients are a commom phenomenon. Three mechanisms have been proposed [7] for them increasing significantly during exercise: 1) increase in non-obstructive physiologycal gradients; 2) end-systolic obstruction secondary to ventricular cavitary obliteration; and 3) mid-systolic obstruction caused by SAM of the mitral valve restricting ejection. However for SAM to occur, there must be some alteration of the geometry of the ventricular chamber or of the mitral valve apparatus. This was not the case in our adolescent, although it has been demonstrated that intraventricular gradients can be caused by maneuvers that change loading conditions in structurally normal hearts [8], and it is know that participating in sports can bring about elicit such changes.

Sudden death in young athlets has been thoroughly studied, and there is an agreement that the usual causes are hereditary or congenital [9]. However, in some series [10] around 30% of autopsy studies show no abnormalities, which suggests that the standard screening programs are failing to prevent sudden death.

In the case we report the fact that the patient had normal echocardiogram means that morphological study of this heart would probably reveal no abnormalities. The phenomenom that we detected *during *exercise testing – mitral valve SAM and intraventricular gradient associated with ST alterations – could well have been responsible for his symptoms.

The medical examination of this athlete was carried out because of symptoms arising from intense effort; we did'nt reproduced the symptoms during exercise test but we detected an anomaly in cardiac function [2-5], that in our opinion may explain them. The abnormality detected is, however, only detectable during effort, and is not among the diagnosis that contraindicate participation in competitive sport according to recommendations of the 36^th ^Bethesda conference [11] and the European Society of Cardiology [12]. It is possible tht the phenomenon described in this adolescent could be among the causes of sudden death in cases where anatomopathological examination reveals no abnormalities, and we accordingly advised the athlete to suspend sports practice and referred him for assessment to a sports medicine center. In our opinion, the case described, in which significant abnormalities in cardiac function were found only during exercise, suggests that this methodology should be applied to other adolescents that have symptoms related to exercise but no structural abnormalities. We should note that this phenomenon has been almost excluded to be a normal response to exercise in young healthy adults [13]. In face of this, the symptomatic athletes in which the findings which we described is detected, should also be subjected of long term follow-up to obtain an accurate assessment of its clinical significance.

## Supplementary Material

Additional file 1 Apical 4 chamber view before exercise. Two-dimensional 4 chamber view, before exercise, showing normal morphology and funtion of left ventricle and mitral valve. Click here for file

Additional file 2 Apical 4 chamber view at 13 minutes before end of exercise. Two-dimensional 4 chamber view, near peak exercise, showing clearly, systolic anterior movement of the mitral valve. Click here for file
